# Identification of the first highly selective inhibitor of human lactate dehydrogenase B

**DOI:** 10.1038/s41598-021-00820-7

**Published:** 2021-11-01

**Authors:** Sachio Shibata, Satoshi Sogabe, Masanori Miwa, Takuya Fujimoto, Nobuyuki Takakura, Akihiko Naotsuka, Shuji Kitamura, Tomohiro Kawamoto, Tomoyoshi Soga

**Affiliations:** 1 Discovery Biology, Discovery Science, Axcelead Drug Discovery Partners, Inc., 2-26-1 Muraoka-Higashi, Fujisawa, Kanagawa Japan; 2 Chemistry, Discovery Science, Axcelead Drug Discovery Partners, Inc., 2-26-1 Muraoka-Higashi, Fujisawa, Kanagawa Japan; 3 Institute for Advanced Biosciences, Keio University, 246-2 Mizukami, Kakuganji, Tsuruoka, Yamagata Japan

**Keywords:** X-ray crystallography, Enzyme mechanisms, Cancer metabolism, High-throughput screening

## Abstract

Lactate dehydrogenase (LDH) catalyses the conversion of pyruvate to lactate and NADH to NAD^+^; it has two isoforms, LDHA and LDHB. LDHA is a promising target for cancer therapy, whereas LDHB is necessary for basal autophagy and cancer cell proliferation in oxidative and glycolytic cancer cells. To the best of our knowledge, selective inhibitors for LDHB have not yet been reported. Here, we developed a high-throughput mass spectrometry screening system using an LDHB enzyme assay by detecting NADH and NAD^+^. As a result, we identified a small-molecule LDHB selective inhibitor AXKO-0046, an indole derivative. This compound exhibited uncompetitive LDHB inhibition (EC_50_ = 42 nM). X-ray crystallography revealed that AXKO-0046 bound to the potential allosteric site away from the LDHB catalytic active site, suggesting that targeting the tetramerisation interface of the two dimers is critical for the enzymatic activity. AXKO-0046 and its derivatives can be used to validate LDHB-associated pathways in cancer metabolism.

## Introduction

Cancer cells can reprogram various genes to promote their rapid proliferation and metastatic potential^1^. Unlike most normal cells, cancer cells can adapt to various microenvironments, such as hypoxia, glucose and other nutrient deficiencies, and acidosis^1^. Moreover, in tumours and other proliferating or developing cells, a metabolic switch from normal oxidative phosphorylation to aerobic glycolysis is common^1^. This adaptation, known as the Warburg effect, allows cancer cells to produce ATP from glucose by promoting glycolysis to produce lactate from the mitochondrial pyruvate pool, even in the presence of oxygen^1,2^. Increased aerobic glycolysis provides cancer cells with a growth advantage, despite its energetic inefficiency compared with oxidative phosphorylation^3^.

During aerobic glycolysis, the conversion of pyruvate to lactate is mediated by cytosolic lactate dehydrogenase (LDH) enzymes with nicotinamide adenine dinucleotide (NADH) as a cofactor, which is converted to nicotinamide adenine dinucleotide (NAD^+^). The LDH enzymes are tetrameric enzymes comprising two separate subunits, M and H, and can form five isozymes, namely, the LDH1 (4H), LDH2 (3H, 1M), LDH3 (2H, 2M), LDH4 (1H, 3M), and LDH5 (4M) subunits. LDH1 and LDH5 are transcribed from the *LDHB* and *LDHA* genes, respectively^4^.

Previous studies have reported that LDHA (LDH5) plays a critical role in cancer. For example, in patients with gastric cancer or non-small-cell lung cancer, high LDHA levels are correlated with tumour size and poor prognosis^5,6^. In addition, LDHA silencing by small interfering RNA or short hairpin RNA inhibits cell growth and tumorigenic potential both in vitro and in xenograft models^7,8^. Therefore, several LDHA inhibitors have been developed whose hit target compounds have been identified through high-throughput screening (HTS)^9^.

LDHB (LDH1) is associated with aggressive cancer phenotypes^10,11^. One study used clinical samples derived from patients with colorectal cancer and found that *MYC* expression is highly correlated with the expression of various metabolic genes. In that study, 231 unique metabolic genes were identified, and the LDHB levels were upregulated, whereas the LDHA levels remained unchanged in colorectal cancer^12^. Furthermore, LDHB is a key contributor to lysosomal activity and autophagy in cancer^13^. Because various cancer cells upregulate autophagy, which is required to support metabolism, tumourigenesis, and resistance to therapy^14^, inhibition of LDHB could be an excellent target for the prevention and treatment of several cancers. Moreover, LDHB is differentially expressed in triple-negative breast cancers, defined by the absence of detectable estrogen receptor and progesterone receptor expression and the lack of human epidermal growth factor 2 gene amplification^11^. *LDHB* knockdown selectively reduces the proliferation of breast cancer cells, both in vitro and in vivo^11^. Triple-negative breast cancer cells exhibit enhanced glycolysis and utilise pyruvate or lactate as an energy source^11^. Therefore, we hypothesised that the inhibition of LDHB may be a promising therapeutic approach for targeting cancer metabolism.

Despite the suggested association between LDHB and cancer metabolism, LDHB-selective inhibitors remain unexplored. In this study, to identify selective LDHB inhibitors, we developed a screening system to monitor the conversion of NADH to NAD^+^ by LDHB using the RapidFire-Mass (RF-MS) system. This assay system was robust and avoided false-positive results. We then identified that AXKO-0046, an indole derivative, selectively inhibited LDHB activity in an uncompetitive manner with respect to NADH and pyruvate. Moreover, structural analysis showed that AXKO-0046 did not bind in the catalytic site of the enzyme but significantly bound to LDHB in the novel allosteric site of the tetramer. The allosteric site was unique to LDHB based on sequence alignment, resulting in LDHB-selective inhibition. Overall, our findings suggest that AXKO-0046 may be a promising chemical probe to elucidate the role of LDHB-associated pathways in cancer metabolism.

## Results

### Development and optimisation of RF-MS for LDHB activity

**Figure 1:**
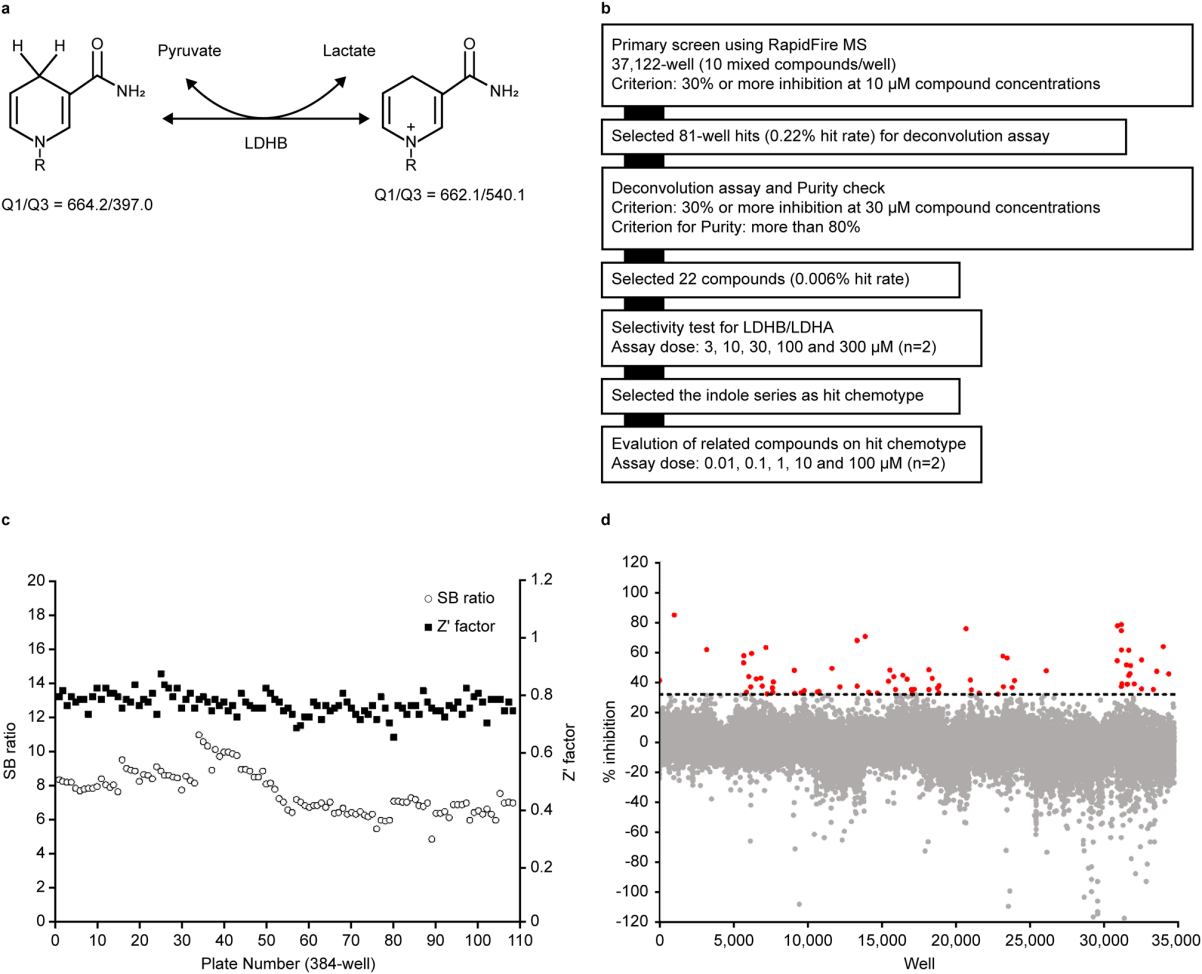
Primary screening for inhibitors of lactate dehydrogenase B (LDHB). (**a)** LDHB-catalysed reaction scheme. NADH and NAD^+^ were detected using mass spectrometry, and their respective MRMs are shown. (**b)** Screening cascade and the results of primary screening. The high-throughput screening cascade consisted of the primary screening, profiling assay, hit validation, and characterisation. A diverse library of small compounds from Axcelead at 10 µM was screened against LDHB. (**c)** SB ratio (open circles) and Z’-factor values (filled squares) of the overall primary screening. (**d)** Scatterplots of primary screening data and selection of hit wells exhibiting greater than 30% inhibitory activity (red circles).

To identify small-molecule LDHB inhibitors, we developed an assay system in a 384-well format to measure LDHB activity through NADH and NAD^+^ by RF-MS (Fig. 1a). The integrated peak areas were linear for concentrations of the NADH or NAD^+^ standard between 1 and 100 μM (Supplementary Fig. 1). The extracted ion chromatogram of NADH also showed a NAD^+^ fragment originating from the in-source decay (ISD) of NADH during ionisation. Because the ISD of NAD^+^ observed with NADH was less than 10% of the peak area of NADH, there was no contribution to the detection of LDHB activity.

Next, we optimised the assay conditions of RF-MS. Titration of human LDHB was examined at concentrations ranging from 0.0625 to 1 nM. Supplementary Fig. 2 shows a linear relationship between the velocity and enzyme concentration (0.0625–0.5 nM). We used 0.25 nM LDHB throughout assay development and screening to ensure enzyme activation. Next, the concentrations of NADH and pyruvate were optimised. The K_m_ values of NADH and pyruvate were 64 and 116 μM, respectively, in the presence of 500 μM pyruvate and increasing concentrations of NADH or 500 µM NADH and increasing concentrations of pyruvate (Supplementary Fig. 2). The K_m_ values obtained from the two assays were consistent with the reported values for LDHB^15,16^. Based on the K_m_ of each substrate and the robustness of the assay signal, NADH and pyruvate concentrations were both set at 75 μM for screening.

### HTS of LDHB inhibitors using RF-MS

The screening campaign was performed on an in-house pooled diverse library of more than 345,000 compounds at a concentration of 10 μM, as shown in the workflow (Fig. 1b). Assay quality was acceptable because Z’ factors exceeded 0.65 and a signal-to-noise ratio greater than 5.0 was obtained (Fig. 1c). The hit rate was 0.22% when the threshold was set at greater than or equal to 30% of inhibition compared with the control (Fig. 1d).

To identify the hit compounds in pooled samples, deconvolution assays were conducted at a compound concentration of 30 μM. Compounds containing oxalate salt, which had inhibitory activities toward LDH, were excluded as undesirable inhibitors because oxalate and its derivatives show only moderate potency and selectivity^17,18^. Thus, 22 compounds showed activity (greater than or equal to 30% inhibition rate and greater than or equal to 80% purity), corresponding to a hit rate of 0.006%. Subsequent dose–response experiments determined the EC_50_ values for all identified hit compounds.

### Comparison of the EC_50_ Values of hit compounds for LDHB and LDHA

To compare the selectivity potency of the 22 selected compounds, we evaluated the compounds against human LDHB and LDHA by RF-MS. To obtain comparable EC_50_ values, the substrate concentration was fixed to each K_m_ determined using our assay conditions for each substrate pair. For LDHA, time-dependent accumulation of NADH was confirmed in a protein concentration-dependent manner (Supplementary Fig. 2). The K_m_ value was also determined for the LDHA reaction. All EC_50_ values are listed in Supplementary Table 1.

Four compounds (i.e., AXKO-0004, AXKO-0008, AXKO-0010, and AXKO-0013) exhibited significant differences in potency (EC_50_) between LDHB and LDHA. Although AXKO-010 was a partial inhibitor at the highest compound concentration, this was not due to compound concentration effects, such as aggregation, instability, and assay interference. Moreover, this compound exhibited LDHB inhibitory activity, with an EC_50_ of less than or equal to 3 μM, but no inhibitory activity against LDHA at 300 μM, thus showing greater than 100-fold selectivity over LDHA (Supplementary Table 1).

**Figure 2:**
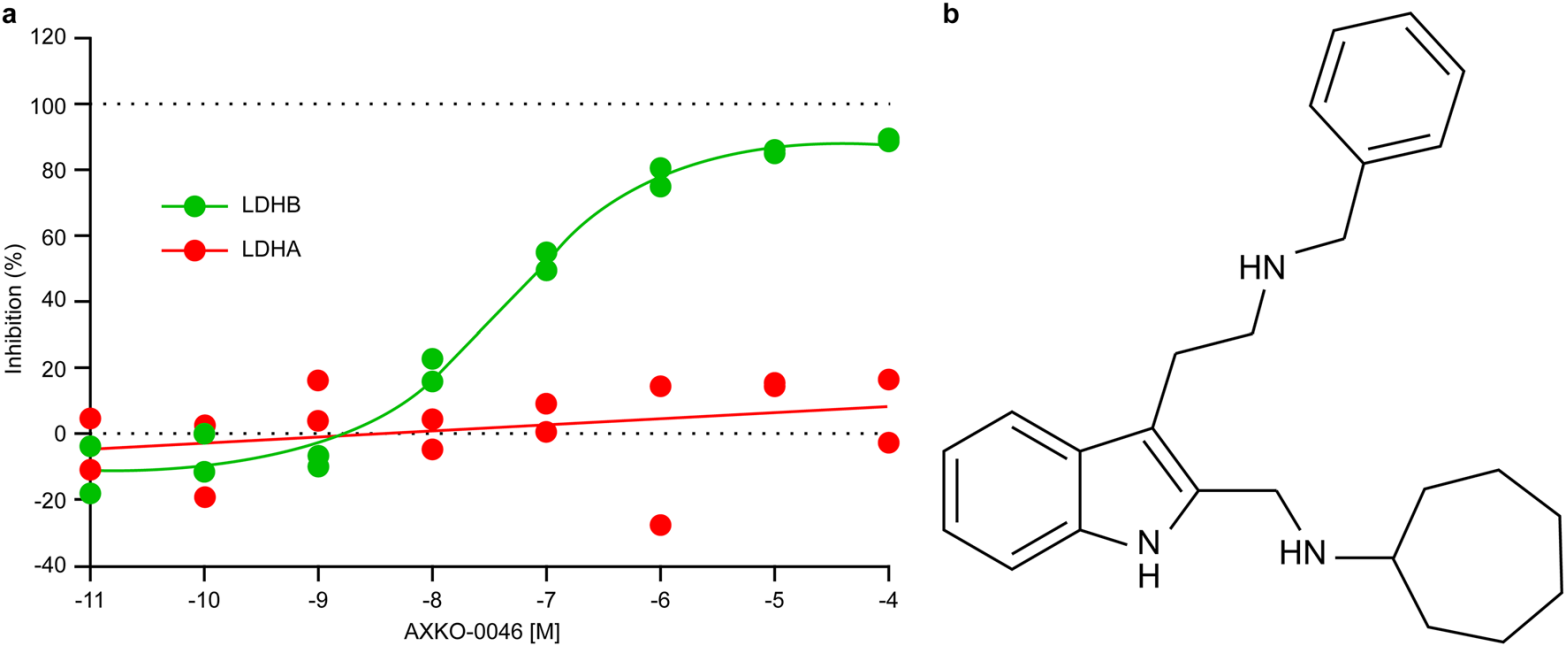
Concentration response curve and the structure of AXKO-0046. (**a)** Concentration response curves and EC_50_ values of AXKO-0046. AXKO-0046 selectively inhibited LDHB (green circles) but did not inhibit LDHA (red circles). The EC_50_ for LDHB was 42 nM. The curves were fitted to the standard four-parameter logistic equation. (**b)** Structure of AXKO-0046.

To identify more potent compounds, we next assessed the inhibitory effects of 75 indole derivatives with substructural similarity from the compound library. AXKO-0046 was identified as N-({3-[2-(benzylamino) ethyl]-1H-indol-2-yl} methyl) cycloheptanamine and achieved highly potent LDHB inhibitory activity (EC_50_ = 42 nM) with selectivity over LDHA (Fig. 2).

### Characterisation of AXKO-0046

**Figure 3:**
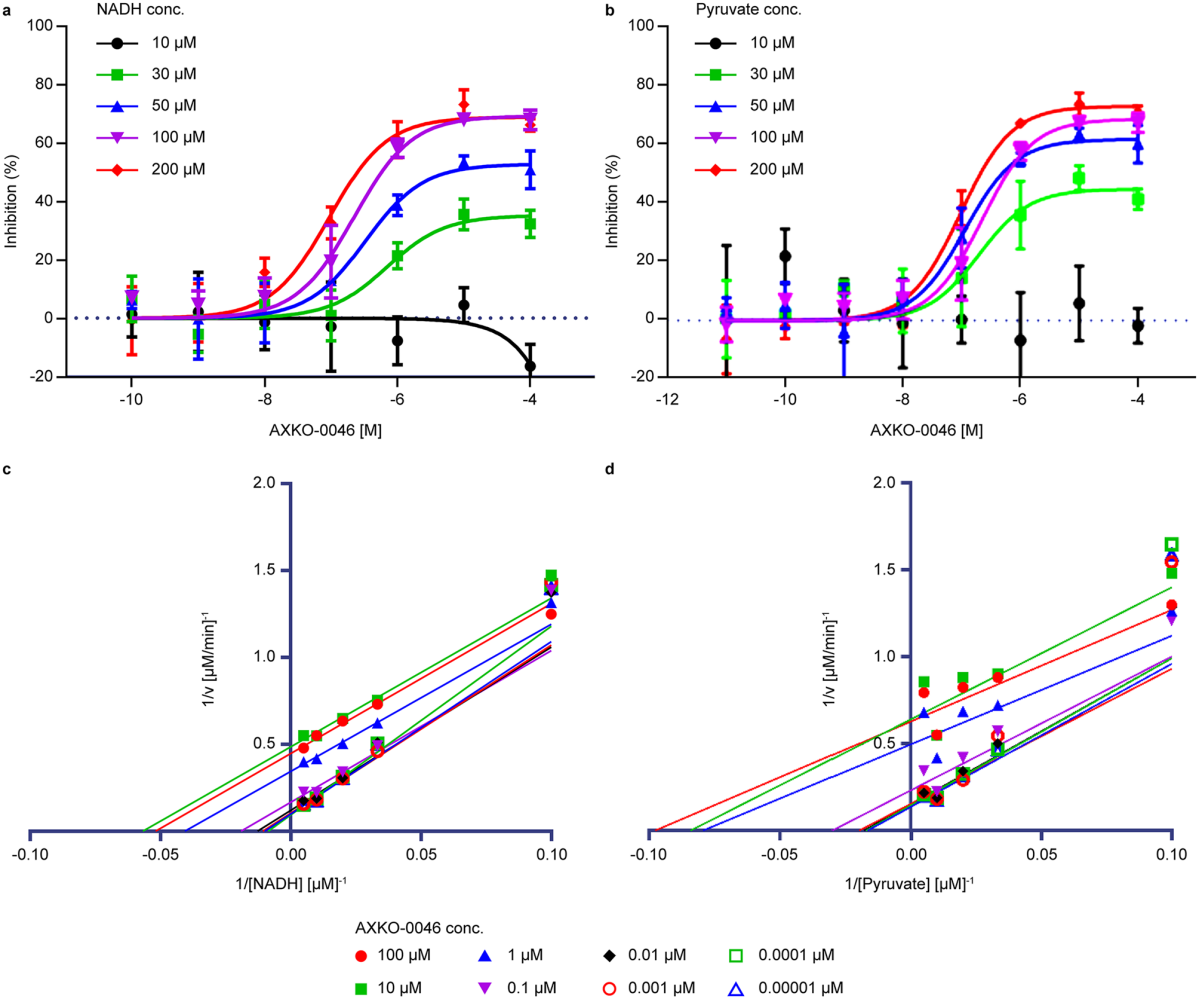
Biochemical characterisation of AXKO-0046. (**a,b)** LDHB inhibition by AXKO0046 was studied using varying concentrations of (**a**) NADH and (**b**) pyruvate. The EC_50_ values of AXKO-0046 decreased with increasing pyruvate and NADH concentrations. Data shown are mean ± standard deviation (n = 3). (**c,d)** Lineweaver–Burk plots of the kinetic data in Table 1 with nonlinear regression analysis of (**c**) NADH and (**d**) pyruvate.

To elucidate the underlying inhibitory mechanism, substrate competition assays were performed. LDHB inhibitory activity for AXKO-0046 was assessed at different concentrations of the substrate. The inhibitory activity of AXKO-0046 increased depending on the concentration of NADH. Similar to NADH, AXKO-0046 exhibited decreasing EC_50_ values at increasing pyruvate concentrations, indicating that it was uncompetitive with both substrates (Fig. 3a,b, and Supplementary Table 2).

Moreover, the K_m_ and V_max_ values for LDHB were determined in the presence AXKO-0046. As the AXKO-0046 concentration increased in the reaction, both V_max_ and K_m_ decreased proportionally (Supplementary Fig. 3 and Table 1). The Lineweaver–Burke plot lines were nearly parallel (Fig. 3c,d), indicating uncompetitive inhibition with a strong preference for inhibiting the enzyme–substrate complex.

**Table 1:** Effects of AXKO-0046 on K_m_ and V_max_ of NADH and pyruvate on LDHB enzyme activity.

	AXKO-0046 concentration [µM]							
	0.00001	0.0001	0.001	0.01	0.1	1	10	100
**NADH**								
V_max_ (μM/min)	10.1	10.7	9.7	8.3	6.0	2.9	2.1	2.2
SD ±	0.1	0.8	0.8	0.7	0.5	0.1	0.1	0.1
K_m_ (μM)	100.4	116	93.5	78.4	52.5	24.6	17.6	19.3
SD ±	20.5	17.4	15.7	15.3	10.7	2.3	2.1	3.0
**Pyruvate**								
V_max_ (μM/min)	7.3	6.9	6.5	6.6	4.3	2.0	1.6	1.6
SD ±	0.9	0.7	0.9	0.8	0.7	0.2	0.1	0.1
K_m_ (μM)	60.3	58.3	50.5	55.2	33.2	12.6	11.9	10.2
SD ±	19.1	15.7	18.8	16.6	16.3	6.4	5.3	4.3

Data shown are mean ± standard deviation (n = 3).

We then assessed whether there was any time dependence to the onset of inhibition by varying the time for which AXKO-0046 and LDHB were pre-incubated before initiating the enzymatic reaction. Following pre-incubation with LDHB for 120 min, the inhibition potential of AXKO-0046 did not increase with pre-incubation time (Supplementary Fig. 4).

### Characterisation of AXKO-0046 derivatives

**Figure 4:**
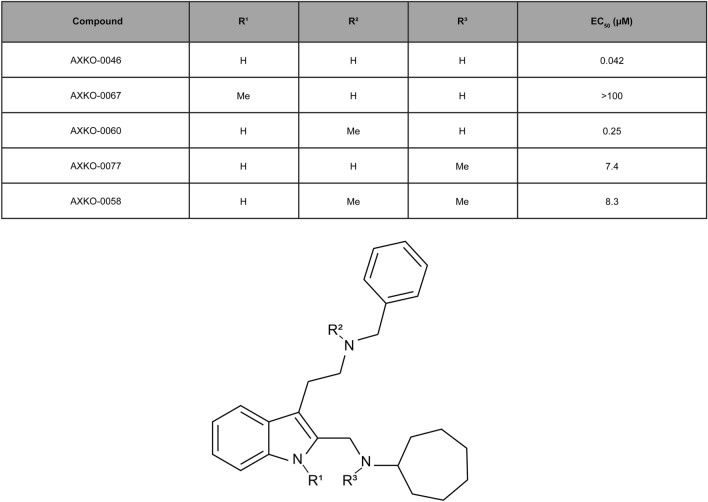
Chemical structure of AXKO-0046 derivatives and their EC_50_ values.

To assess the structure–activity relationships (SARs) of AXKO-0046, its derivatives were synthesised, and their inhibitory activities were evaluated. Methyl scanning is a well-known systematic approach to determine interaction sites between small molecules and macromolecular targets, thus providing opportunities for structural modifications to improve potency^19^. The structure and inhibitory activity of each compound are listed in Fig. 4.

AXKO-0046 contains three hydrogen bond donors, that is, the NH group of indole, benzylamine, and cycloheptylamine, which can interact with the counter amino acid residues of LDHB. The introduction of a methyl group to the benzylamino group (AXKO-0060) resulted in a moderate loss of inhibitory activity (EC_50_ = 0.25 µM), whereas the addition of the methyl group at the cycloheptylamino group (AXKO-0077) resulted in a considerable loss of activity (EC_50_ = 7.4 µM). The incorporation of two methyl groups into both amino groups (AXKO-0058) simultaneously reduced activity (EC_50_ = 8.3 µM). Finally, methylation at the 1-position of indole (AXKO-0067) led to a significant loss of potency (EC_50_ > 100 µM).

### Structural analysis of AXKO-0046 with LDHB

**Figure 5:**
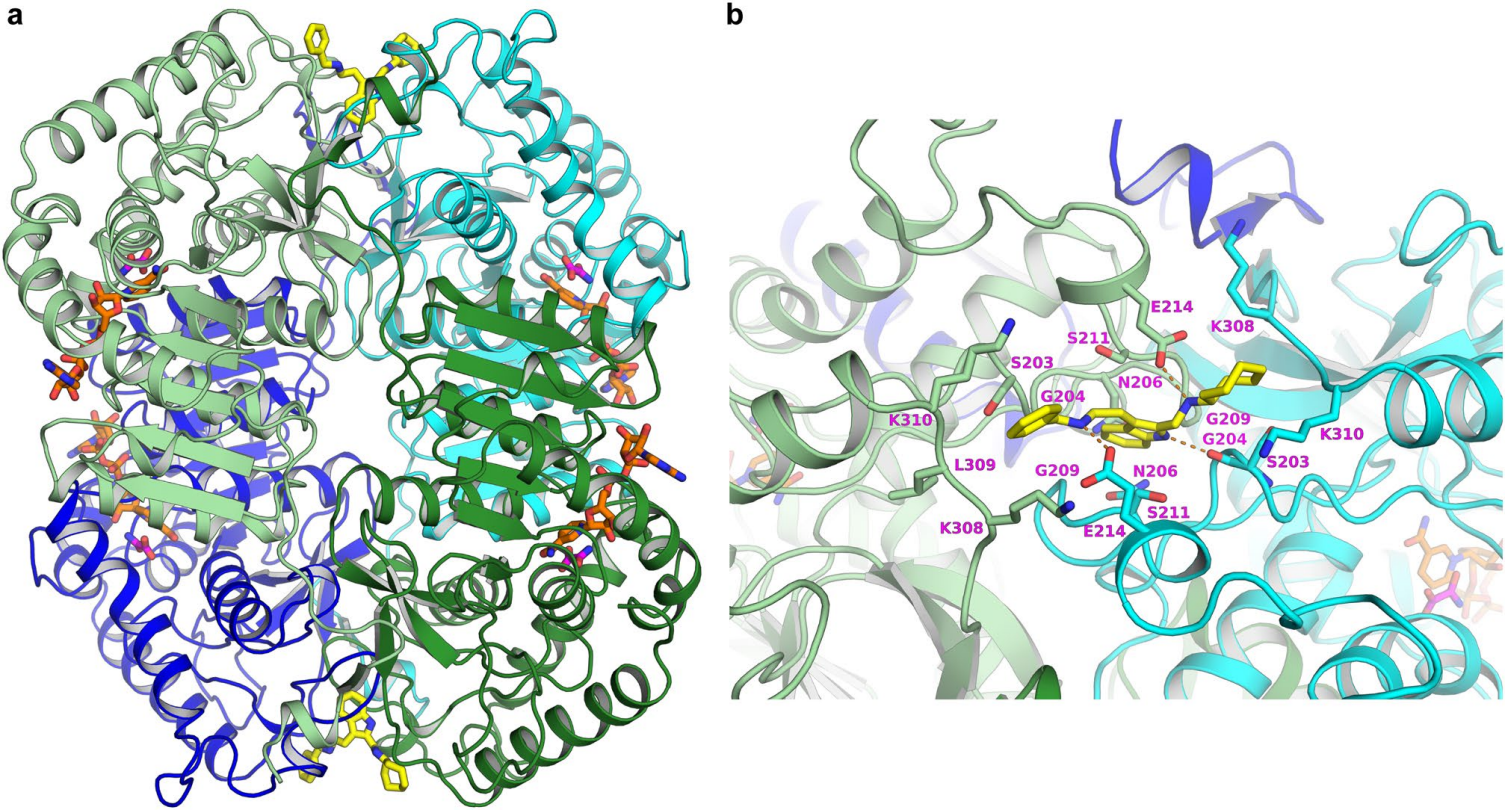
Crystal structure of the quaternary LDHB/NADH/oxamate/AXKO-0046 complex. (**a)** Ribbon diagram. (**b)** Close-up view of the binding site of AXKO-0046. Each monomer of the tetramers is shown in lime, forest, cyan, and blue. NADH, oxamate, and AXKO-0046 are depicted as stick models in orange, magenta, and yellow, respectively.

To investigate the binding site, we solved the two crystal structures of LDHB, that is, the binary complex with the cofactor NADH and the quaternary complex with NADH using the substrate analogue oxamate and the inhibitor AXKO-0046 at 1.80 and 1.55 Å resolution, respectively (Fig. 5a and Supplementary Table 3). Both structures exhibited α/β protein folding conserved in the LDHA and LDHB structures, as previously reported^20^. There were two tetramers for the binary complex and one tetramer for the quaternary complex in the asymmetric unit. The average root-mean-square deviation (RMSD) values between the monomers in the asymmetric unit were 0.5 and 0.3 Å for two tetramers of the binary complex and one tetramer of the quaternary complex, respectively. Electron density was visible for NADH and oxamate at the cofactor- and substrate-binding sites, respectively, whereas relatively weak but distinct electron density was visible for AXKO-0046 at the interface between the two monomers (Supplementary Fig. 5). These results indicated that the compound had alternative conformations on the two-fold axis of the tetramer with relatively low occupancies.

The binding conformation of NADH with the enzyme was essentially identical between the binary complex and the quaternary complex, and their binding interactions were consistent with those of published structures^20–22^. In the quaternary complex, oxamate was bound near the NADH nicotinamide moiety and interacted with the active-site loop (residues Glu101–Leu110), which was in a closed conformation. In contrast, in the binary complex, the active-site loops were partially disordered with an open conformation for the two tetramers in the asymmetric units, except for two of eight monomers in which the loop was well ordered owing to crystal packing (Supplementary Fig. 6). The superimposition of our two structures clearly indicated structural changes corresponding to AXKO-0046 inhibition. Significant structural differences were observed around the active-site loop, with a maximum RMSD of approximately 7 Å for the main-chain atoms. Other marked structural deviations, except for the active site, were observed in the two helices near the active site (residues Trp228–Lys246 and residues Asp311–Asp330), with a maximum RMSD of approximately 2 Å for backbone atoms (Supplementary Fig. 7). These structural variations are further described in the Discussion to elucidate the inhibitory mechanism of AXKO-0046.

AXKO-0046 was located in an interfacial allosteric site between two monomers, approximately more than 20 Å away from the active site (Fig. 5b). Although the compound occupied the site with half-occupancy in the two possible orientations on a two-fold axis, only one orientation was defined owing to the relatively poor electron density, as described in Supplementary Fig. 5. The indole ring thus occupied the small cavity composed of Gly204, Asn206, Gly209, and Ser211. In addition, the NH group of indole formed a hydrogen bond with the main-chain oxygen of Ser203. Two NH groups of benzylamine and cycloheptylamine interacted with the side-chain oxygens of two Glu214 residues. The benzyl and cycloheptyl groups were exposed to the solvent region and surrounded by Lys308 and Lys310, followed by the C-terminal helix with significant structural rearrangements, as described below.

## Discussion

Several metabolic enzymes play major roles in cancer survival; however, they are not extensively exploited as drug targets. Therefore, in this study, we utilised LDHB as a drug target and developed an assay for high-throughput compound screening to identify LDHB inhibitors. To the best of our knowledge, LDHB-specific inhibitors have not been reported owing to the high structural homology between LDHB and LDHA and the high structural homology of their catalytic sites (89% according to the NCBI Basic Local Alignment Search Tool [BLAST])^23^. Furthermore, high-throughput screening for the identification of LDHB-specific inhibitors has not yet been reported.

The metabolic model of lactate shuttling in the tumour microenvironment is known as metabolic symbiosis in tumours^24,25^, where LDHB is a key molecule of the oxidative pathway of lactate that controls metabolic symbiosis between glycolytic and oxidative cancer cells^26,27^. In oxidative cancer cells, lactate is imported by MCT1, located in the cell plasma membrane, and LDHB oxidizes lactate to pyruvate to produce energy. Comparatively, glycolytic cancer cells generate lactate from pyruvate via the LDHA reaction. LDHB is needed to sustain glycolytic cancer cell survival. In other words, targeting lactate in oxidative cancer cells could offer a unique opportunity to induce necrosis of distant glycolytic cancer cells known to be resistant to conventional antitumor treatments^26^. Therapeutic strategies to induce necrosis in glycolytic cancer via the inhibition of lactate transport are currently being applied in early clinical trials for cancer treatments using the MCT-1-selective inhibitor AZD3965^28^. AZD3965 is in the early phase clinical trials (www.clinicaltrials.gov) with phase I expansion cohort enrichment for Burkitt lymphoma and diffuse large B cell lymphoma cases. Moreover, studies have indicated that complete hereditary deficiency of LDHB has no symptomatic consequences in humans^29,30^. On the contrary, patients with hereditary LDHA deficiency have been reported to present with exertional myopathy, erythematosquamous skin lesions, and uterine stiffness during pregnancy^31,32^. Therefore, these results indicate the possibility of the future clinical development of pharmacological inhibitors of LDHB with high efficacy and safety.

To date, in vitro LDH assays have been used to measure the fluorescence of NADH, with a characteristic excitation maximum at 340 nm and an emission maximum at 480 nm^32,33^. However, this procedure can give rise to false positives and negatives because of fluorescence interference at the excitation and emission wavelengths of NADH. Although other methods, such as measuring NADH through the conversion of resazurin to resorufin by diaphorase using enzyme-coupled detection methods^9^, have also been suggested, inhibition of the coupling enzymes by test compounds may affect the assay and lead to false positives in HTS campaigns.

Therefore, the Genentech group developed a label-free assay to monitor the conversion of pyruvate to lactate by LDHA using RF-MS^34^. However, this approach was only used for secondary assays to confirm the selected compounds from initial screening employing fluorescence assay. In this study, instead of measuring the conversion of pyruvate to lactate, we established a robust RF-MS assay system to monitor that from NADH to NAD^+^ using LDH activities. This method may be useful not only for LDH but also for other dehydrogenases utilizing NADH or NAD^+^. Additionally, this approach allowed us to conduct HTS campaign using a large compound library. The assay showed excellent performance, with an average Z’ factor greater than or equal to 0.65 when more than 345,000 compounds were screened.

The hit rate in the screening campaign was very low (0.006% with 30% inhibition threshold). This low hit rate corresponded to the data when screened against LDHA using a small compound library^34^. LDHA is considered a highly intractable target against small molecules^18^. However, we found 22 substantially potent LDHB inhibitors, of which only four compounds (i.e., AXKO-0004, AXKO-0008, AXKO-0010, and AXKO-0013) showed high selectivity for LDHB after retesting at five concentrations. Among these hit compounds, AXKO-0010, which contained a central indole scaffold, showed excellent potency and selectivity as a LDHB inhibitor. However, this compound did not completely inhibit enzyme activity, even after an increase in the concentration. Therefore, we next assessed the inhibitory effects of a subfamily of structurally related compounds to AXKO-0010. We found that 63 of 75 related compounds showed highly potent LDHB inhibitory activities while maintaining selectivity. Of these, AXKO-0046 showed the greatest potency for LDHB inhibition (EC_50_ = 42 nM).

Next, the mechanism of LDHB inhibition of AXKO-0046 was evaluated using substrate-competition assays. AXKO-0046 was tested at five different NADH and pyruvate concentrations. Unexpectedly, increasing the concentration of pyruvate and NADH correlated with increased AXKO-0046 inhibitory activity, suggesting an uncompetitive inhibitory mechanism with respect to both NADH and pyruvate. These findings indicated that this compound likely bound to the enzyme–substrate complex. Several other selective LDHA inhibitors, such as galloflavin, oxamate, diacid-malonate scaffolds, and quinoline 3-sulphonamides, have been described; however, these compounds are substrate-competitive inhibitors owing to the interaction with the catalytic site^9,35–37^.

Notably, the NADH concentration in cancer cells is generally elevated and has been reported to range from 168 to 870 μM^38^. LDHA likely binds to NADH in cells. Thus, a competitive inhibitor of NADH may yield poor cellular activity^37^. Because AXKO-0046 is uncompetitive with respect to both NADH and pyruvate, we hypothesise that increased intracellular NADH and pyruvate concentrations may increase enzyme inhibition, unlike competitive behaviour. Structural studies provide further insights into the binding site of AXKO-0046 and the interaction residues of the LDHB protein for compound design to inhibit LDHB activity.

The crystal structure of the quaternary complex of LDHB with NADH, oxamate, and AXKO-0046 revealed that AXKO-0046 was an allosteric inhibitor distant from the catalytic site. The binding mode of AXKO-0046 spatially correlated with SAR studies to rationalise critical functional groups. The indole ring occupied a shallow pocket between the dimer interface, and its NH group formed a hydrogen bond with the main chain oxygen of Ser203. The reduced LDHB inhibitory activity of the N-Me derivative (AXKO-0067) was attributed to the disappearance of the interaction. In addition, two amino groups of AXKO-0046 could form hydrogen bonds with Glu214 at the symmetry-related location of the tetramer. The methylated derivatives of AXKO-0046 (AXKO-0060, AXKO-0077, and AXKO-0058) also showed reduced LDHB inhibitory activity, which may be related to the attenuation of the interactions. The glutamic acid of LDHB (Glu214) was replaced by threonine in LDHA (Supplementary Figures S8 and S9). Near the AXKO-0046 binding site, two lysine residues of LDHB (Lys308 and Lys310) were also substituted by threonine in LDHA. Thus, the selectivity of AXKO-0046 may be attributed to these amino acid differences.

There are two binding sites in the tetramer that are located at the interface of two dimers. In bacterial LDH enzymes, the allosteric site of the activator, fructose 1,6-bisphosphate (FBP), is involved in the regulation of enzymatic activity^39^. The FBP-binding site is also located at the dimer interface but is structurally distinct from the binding site of AXKO-0046. Moreover, the regulatory mechanism of bacterial LDHs is not applicable to the inhibitory mechanism of AXKO-0046 against LDHA and LDHB. Based on the crystal structures, the allosteric transition by the activator within two subunits is not required for the LDHB activity. Machilin A (MA) has been reported to bind to LDHA in an interfacial allosteric site close to the FBP binding site^40^. However, its binding conformation of MA remains controversial because steric clashes of the protein–ligand interactions have been observed. Recently, selective allosteric inhibitors of the human LDHA isoenzyme with submicromolar activity in vitro^41^. Two compounds with selectivity against LDHA are bound to a novel binding site adjacent to the active site. Overall, we concluded that the binding site of AXKO-0046 was a unique allosteric binding site of a selective LDHB inhibitor.

**Figure 6:**
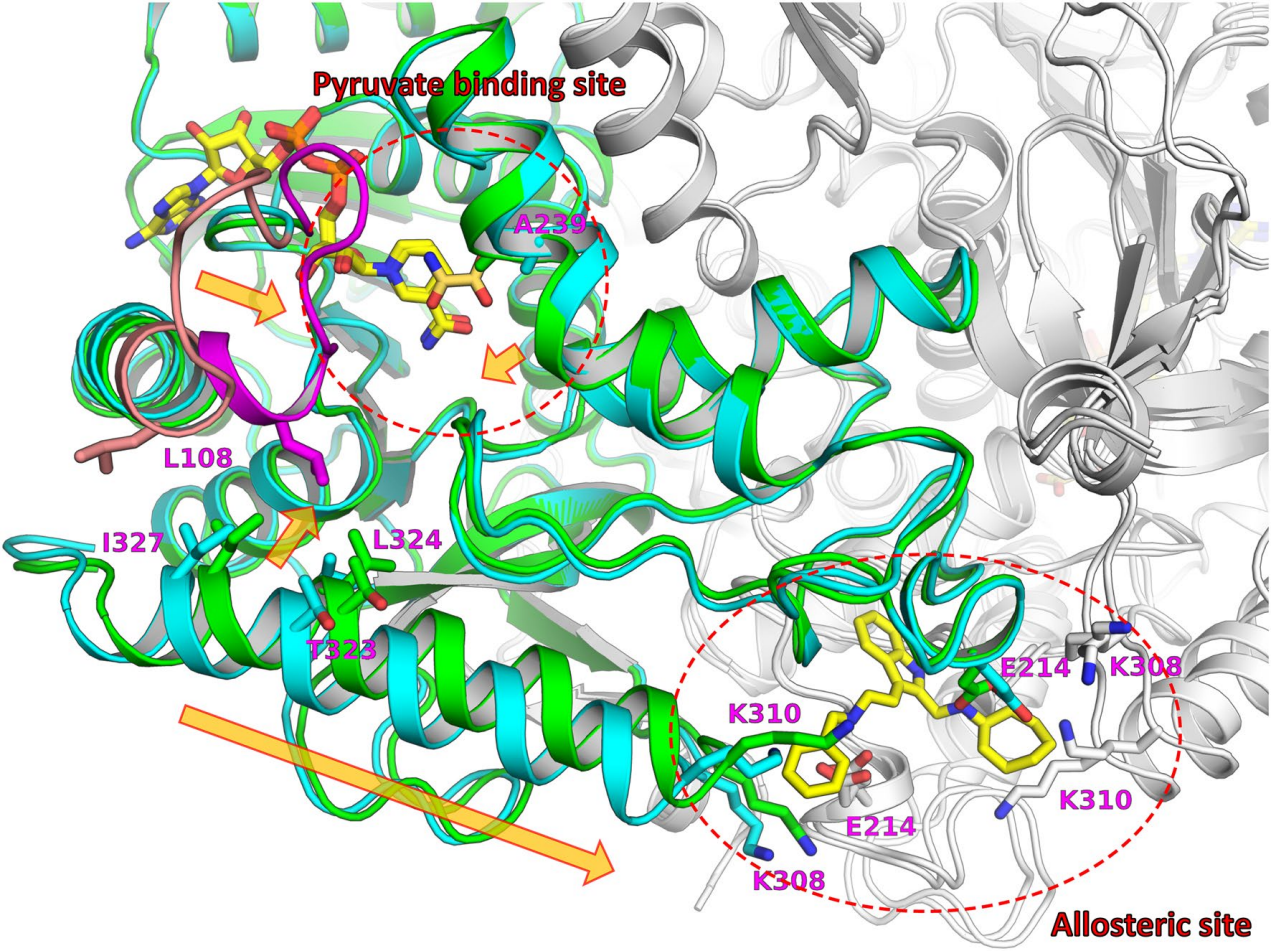
Superposition of the LDHB/NADH/oxamate/AXKO-0046 complex (green or magenta) with the LDHB/NADH complex (cyan or salmon). For clarity, one monomer of each tetramer is coloured, whereas others are in grey. NADH, oxamate, and AXKO-0046 are depicted as stick models in yellow, yellow-orange, and yellow, respectively. Key side chains of key residues are also depicted as stick models and labelled. The active site and allosteric site are highlighted in red dot circles. Structural rearrangements by substrate binding are indicated with orange arrows.

A structural comparison between our quaternary and binary complexes revealed that the active-site loop, referred to as the substrate-specificity loop, exhibited closed and open conformations during the binding and release of substrates, respectively (Supplementary Fig. 7). The active-site loop of the closed conformation interacted with the C-terminal helix through hydrophobic interactions among Leu108, Thr323, Leu324, and Ile327. The substantial conformational changes in the active-site loop, accompanied by the conformational shift in the C-terminal helix, were associated with the binding site of AXKO-0046 (Fig. 6). These results suggested that substrate binding mediated the forging of the allosteric binding site and facilitated the specific binding of AXKO-0046. Consequently, the AXKO-0046 binding interfered with the machinery of the enzyme, although the substrate was bound. This structural observation was consistent with the results of substrate-competition assay suggesting that the inhibitory mechanism of AXKO-0046 was uncompetitive.

In conclusion, the LDHB assay we developed to monitor LDHB activity had numerous advantages over current assay methods. In particular, our method was continuous, nonradioactive, irreversible, robust, and versatile. In this study, we showed that AXKO-0046 acted as a potent specific inhibitor of LDHB in an uncompetitive manner. Thus, AXKO-0046 may be helpful for the exploration of molecular probes to elucidate the biological functions and therapeutic relevance of LDHB as a drug target. The compound may also be a valuable tool to elucidate cancer metabolism. However, AXKO-0046 exhibited no potent cellular activity with clear pharmacodynamic properties. Furthermore, it may be necessary to perform lead optimisation for this series to enable in vivo evaluation at exposure levels that are not generally toxic. Our findings lay a foundation for the development of drugs to treat LDHB-related diseases.

## Methods

### Materials

Recombinant human LDHA and LDHB were purchased from R&D Systems (Minneapolis, MN, USA). NAD^+^ was purchased from MP Biomedicals (Santa Ana, CA, USA). DTT, NaCl, and Triton X-100 were obtained from Sigma-Aldrich (St. Louis, MO, USA). Dihexylammonium acetate (DHAA) was obtained from Tokyo Kasei Kogyo (Tokyo, Japan). NADH, pyruvate, bovine serum albumin (BSA), and other reagents were purchased from Fujifilm Wako (Osaka, Japan).

AXKO-0046 was synthesised by Takeda Pharmaceutical Co., Ltd. (Osaka, Japan); AXKO-0058, AXKO-0060, AXKO-0067, and AXKO-0077 were synthesised by Axcelead Drug Discovery Partners, Inc. (Kanagawa, Japan).

### LDHB and LDHA assays for primary screening

We screened for compounds (final concentration: 10 μM) within pooled libraries consisting of more than 345,000 compounds. Each compound in DMSO (1 mM) was dispensed into 384-well V base plates (100 nL/well) using an Echo 555 acoustic dispenser (Labcyte, Sunnyvale, CA, USA). LDHB (5 μL) was diluted in assay buffer (20 mM Tris [pH 7.5], containing 2 mM DTT, 0.005% Triton X100, and 0.005% BSA) and dispensed into assay plates (final concentration: 0.25 nM). The plates were centrifuged (200× *g* for 1 min) and incubated at room temperature for 30 min, and 5 μL of NADH and pyruvate were added to the assay buffer (final concentration: 75 μM). After incubation for 30 min at room temperature, acetonitrile (10 μL) and water (50 μL) were added to quench the reaction. All additions were performed using a Multidrop Combi (Thermo Fisher Scientific, Waltham, MA, USA). Assay plates were sealed and centrifuged (2700× *g* for 10 min) before storage at − 20 °C.

The conditions for LDHA assays were as described above, except for substrate concentrations, that is, 400 µM pyruvate and 100 µM NADH. These concentrations were chosen based on fitting to the Michaelis–Menten model with the K_m_ values (Supplementary Figure S2).

### High-throughput mass spectrometry

For RF-MS (Agilent, Wakefield, MA, USA), the enzyme reaction solution (5 μL) was aspirated directly from the quenched assay plates and loaded onto a C8 solid-phase extraction cartridge (Agilent) with a mobile phase of water containing DHAA (5 mM) for 2500 ms at a flow rate of 1.25 mL/min. The analytes were then co-eluted into the mass spectrometer using water:acetonitrile:acetone (2:1:1) containing ammonium acetate (5 mM) for 5000 ms at a flow rate of 1.0 ml/min. NADH and NAD^+^ were detected by multiple reaction monitoring with QI/Q3 transitions at *m/z* 664.2 to 397.0 and *m/z* 662.1 to 540.1, respectively, on a Sciex API4000 triple quadrupole mass spectrometer (Applied Biosystems, Foster City, CA, USA) in the negative electrospray ionization mode. The extracted ion chromatograms for each transition were integrated and processed using RapidFire Integrator (Agilent). The data for each well were normalised using the monitoring product conversion with the ratio of AUC_product_ / (AUC_product_ + AUC_substrate_).

To determine the compound activities, percent inhibition data normalised to 0% (DMSO only) and 100% inhibition (no-enzyme control) wells were calculated. Data analysis was performed using the Tibco Spotfire (Boston, MA, USA) packages.

### Substrate-competition assay

To evaluate the mechanism of inhibition of the selected compounds, their initial reaction velocities were measured using five concentrations of NADH or pyruvate (10, 30, 50, 100, and 200 μM) incubated with 100 μM pyruvate for NADH titration or 100 μM NADH for pyruvate titration. A solution of AXKO-0046 (0.00001, 0.0001, 0.001, 0.01, 0.1, 1, 10, and 100 μM) with 0.25 nM LDHB was then added. The enzymatic reactions were performed at room temperature for 15 min in triplicate. At each inhibitor concentration, the dependence of the initial reaction velocity on the substrate concentration was fitted to a nonlinear Michaelis–Menten model to obtain the K_m_ and V_max_ values using GraphPad Prism. Lineweaver–Burk plots were generated by superimposing the data of 1/[velocity] and 1/[substrate] and the line corresponding to the Michaelis–Menten nonlinear fit.

### Protein preparation for structural analysis

Human *LDHB* (Ala2–Leu334; NCBI Reference Sequence: NM_001174097.2) DNA was synthesised and ligated into a pET21a vector (Merck Millipore, Darmstadt, Germany) with an N-terminal His-Avi tag followed by a TEV protease cleavage site using VectorBuilder (Chicago, IL, USA). The expression plasmid was transfected into *Escherichia coli* BL21 (DE3) (Nippon Gene, Toyama, Japan), and the cells were grown in lysogeny broth medium containing ampicillin (100 mg/mL). Protein expression was induced with isopropyl β-d-1-thiogalactopyranoside (0.2 mM) following culture for 16 h at 16 °C. The harvested cells were lysed by sonication in lysis buffer (50 mM Tris, pH 8.0) containing NaCl (150 mM) nuclease (5 U/mL) and centrifuged at 15,000× *g* for 10 min at 4 °C. The clarified supernatant was loaded onto an Ni–NTA cartridge (FUJIFILM Wako, Osaka, Japan), and the eluted fraction was purified on a HiLoad 26/60 Superdex 200 pg column (GE Healthcare, Piscataway, NJ, USA). The His-Avi tag was digested using TEV protease (Sigma-Aldrich), and the digested solution was subsequently passed through an Ni–NTA column to remove TEV protease and the uncleaved protein.

The protein was further purified by anion-exchange chromatography (monoQ; GE Healthcare) with a linear gradient of NaCl. The products of all purification steps were assessed by sodium dodecyl sulfate polyacrylamide gel electrophoresis and Coomassie Blue staining (Sigma-Aldrich). The final yield of protein was 46 mg for every 1 L of culture. For crystallisation, the purified protein was buffer-exchanged to the final buffer (50 mM Tris [pH 7.6] with 150 mM NaCl), concentrated (20 mg/mL) by ultrafiltration (AMICON-ULTRA 10 K; Millipore, Bedford, MA, USA), and stored at − 80 °C.

### X-ray crystallography

The complexes of LDHB with NADH or NADH, oxamate, and AXKO-0046 were generated by incubating threefold molar excesses of ligands on ice for 2–3 h before crystallisation experiments. Both complexes were crystallised from a reservoir solution containing HEPES (0.1 M [pH 7.5]), potassium formate or ammonium acetate (0.2 M), and PEG 3350 (20% v/v) at 20 °C via the sitting-drop vapour diffusion method. Prior to data collection, crystals were immersed in the reservoir solution containing ethylene glycol (30%) as a cryoprotectant and flash-frozen in liquid nitrogen. Diffraction data were collected from a single crystal using a DECTRIS Pilatus3-S6M PAD detector (Baden-Daettwil, Switzerland) with a BL45XU beamline (SPring-8, Hyogo, Japan) under a 100-K nitrogen cryostream. The diffraction data were reduced and scaled using HKL2000^42^.

The structure was solved following the molecular replacement method using Phaser^43^ in the CCP4 software suite^44^ and the LDHB structure (PDB code 1I0Z^18^) as a search model. Refinement was performed using REFMAC5^45^ with individual isotropic restrained B factors. Some data (5%) were set aside for crossvalidation before refinement, and progress was monitored using *R*_free_. For TLS refinement, the tetramer of the protein and ligands was set as a single rigid body^46^. Interactive model building was performed using COOT^47^. The final models were validated using Molprobity^48^. All graphical figures were generated using PyMOL (Schrödinger LLC, Cambridge, MA, USA). Crystallographic processing and refinement statistics are summarised in Supplementary Table S1.

### Data analysis

For the kinetic parameters, the initial rates of LDH activity were determined by incubating LDHA or LDHB with various concentrations of NADH and pyruvate in the assay buffer at room temperature. The reaction product was measured by RF-MS. To estimate the K_m_ and V_max_ values, the initial rates were fit to the Michaelis–Menten equation. EC_50_ and Hill slope calculation and curve fitting using four-parameter fits were performed using GraphPad Prism v6.07 (GraphPad Software, San Diego, CA, USA) or XLfit (IDBS, Guildford, UK) with nonlinear regression analysis, wherein the EC_50_ value equaled the concentration at which the inflection point of the fitted model was reached. The quality and robustness of the screening campaign process were determined by analysing the Z’ factor.

## Supplementary Information

Supplementary Information.

## Data Availability

The atomic coordinates and structure factors have been deposited in the Protein Data Bank with the primary accession codes 7DBJ and 7DBK. The data that support the findings of this study are available from the corresponding authors upon reasonable request.
