# Supplementary material for: Identification of the first highly selective inhibitor of human lactate dehydrogenase B
Source: Sci Rep. 2021 Nov 1;11:21353. doi: 10.1038/s41598-021-00820-7 (PMC8560939; doi:10.1038/s41598-021-00820-7)
Supplement: Supplementary file 1 — Supplementary Information. [file 41598_2021_820_MOESM1_ESM.docx]

**Supplementary Information**

**Identification of the First Highly Selective Inhibitor of Human Lactate Dehydrogenase B**

Sachio Shibata^1†^*, Satoshi Sogabe^1#^, Masanori Miwa^1^, Takuya Fujimoto^2^, Nobuyuki Takakura^2^, Akihiko Naotsuka^1^, Shuji Kitamura^2^, Tomohiro Kawamoto^1^*, and Tomoyoshi Soga^3^

Supplemental Data includes:

Supplementary Figure 1. Linearity of MS-based NADH and NAD+ detection.

Supplementary Figure 2. Determination of LDHB and LDHA assay conditions.

Supplementary Figure 3. Nonlinear fits of Michaelis–Menten kinetics for LDHB in the presence of AXKO-0046.

Supplementary Figure 4. Time-dependent inhibition of LDHB activity by AXKO-0046.

Supplementary Figure 5. Fobs-Fcalc electron density omit maps of the quarterly complex contoured at 3σ.

Supplementary Figure 6. Superposition of eight monomers in the asymmetric unit of the LDHB/NADH complex.

Supplementary Figure 7. Comparison of the root-mean-square deviation values per amino acid residue of LDHB between the two complexes.

Supplementary Figure 8. Sequence alignment of human LDHA and human LDHB.

Supplementary Figure 9. Structural comparison between the allosteric sites of LDHB and LDHA (PDB code 4OKN).

Supplementary Table 1. Selectivity profile of the LDHB hit compound.

Supplementary Table 2. Inhibitory activity of LDHB by AXKO-0046 at varying concentrations of NADH and pyruvate.

Supplementary Table 3. Data collection and refinement statistics.

**
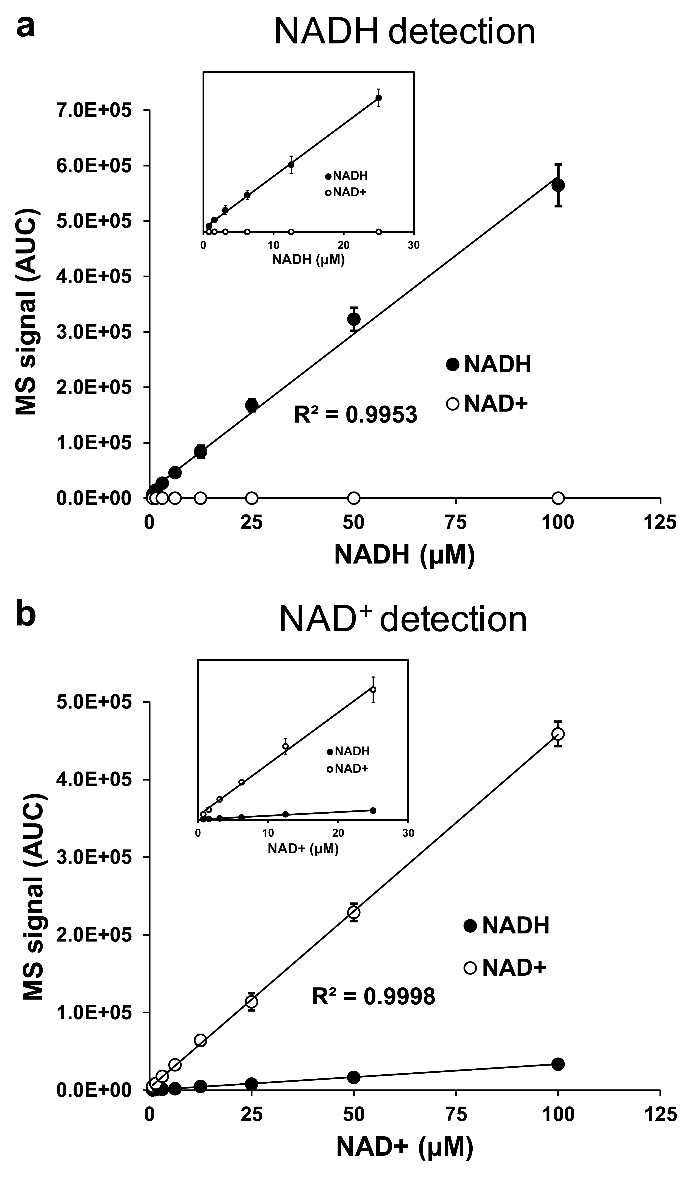
**

**Supplementary Figure 1. Linearity of MS-based NADH and NAD^+^ detection.**

Linearity was detected up to 100 μM NADH (**a**, closed circles) and NAD^+^ (**b**, open circles) with a limit of detection at 0.78 µM using RF-MS. Linearity is shown as insets for lower concentrations of the analytes. Data shown are mean ± standard deviation (n = 4).

**
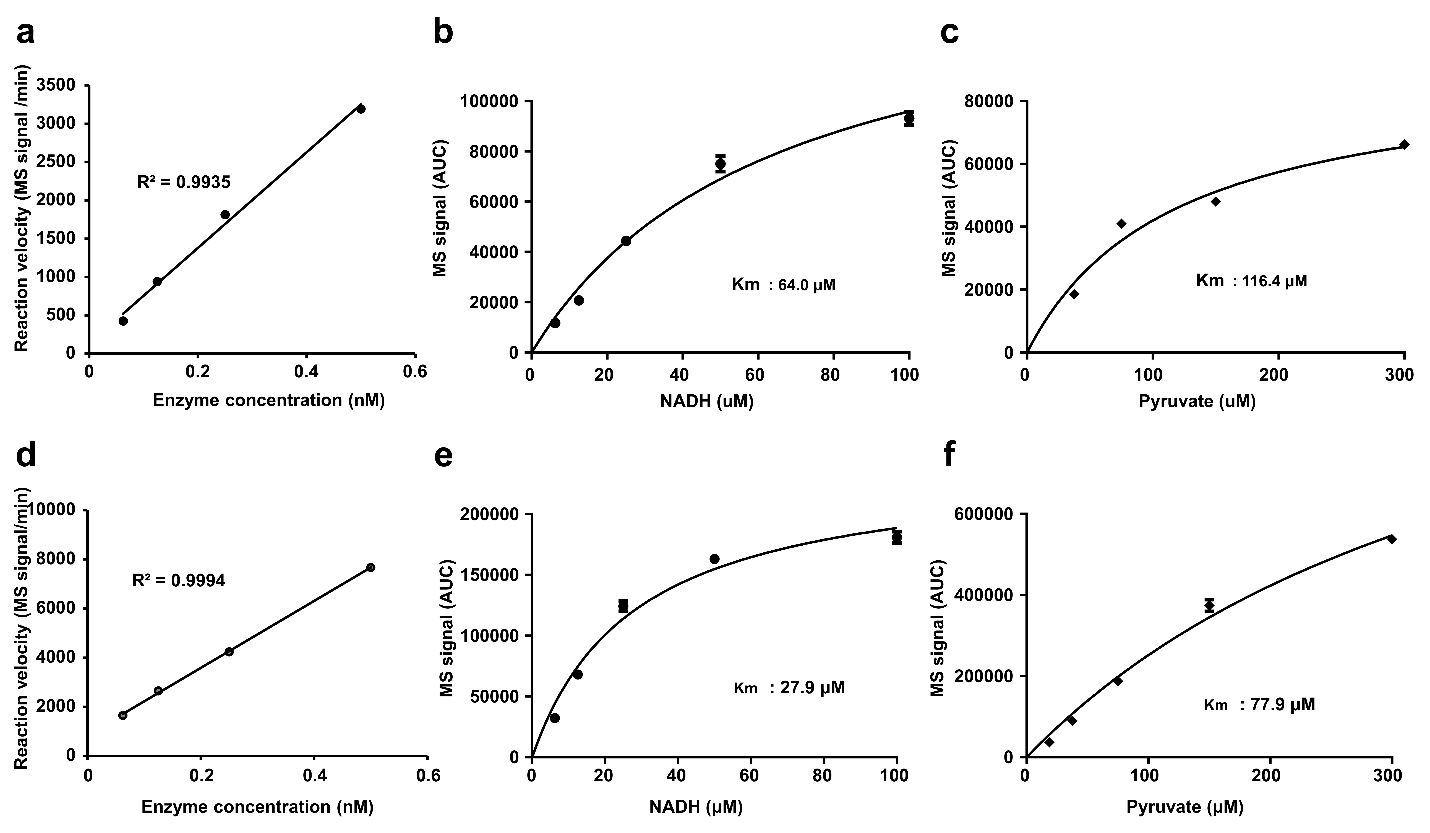
**

**Supplementary Figure 2. Determination of LDHB and LDHA assay conditions.**

**a, d** Initial velocities of LDHB with pyruvate (75 µM) and NADH (75 µM) determined using RapidFire MS. The LDHB or LDHA reaction (0.0625–0.5 nM) showed good linearity up to 20 min. The assay was performed with pyruvate (500 µM) and increasing concentrations of NADH (**b**, **e**) or NADH (500 µM) and increasing concentrations of pyruvate (**c**, **f**). Data were fitted to the Michaelis–Menten equations to obtain the kinetic constant K_m_ using GraphPad Prism. Data are shown as mean ± standard deviation (n = 4).

**
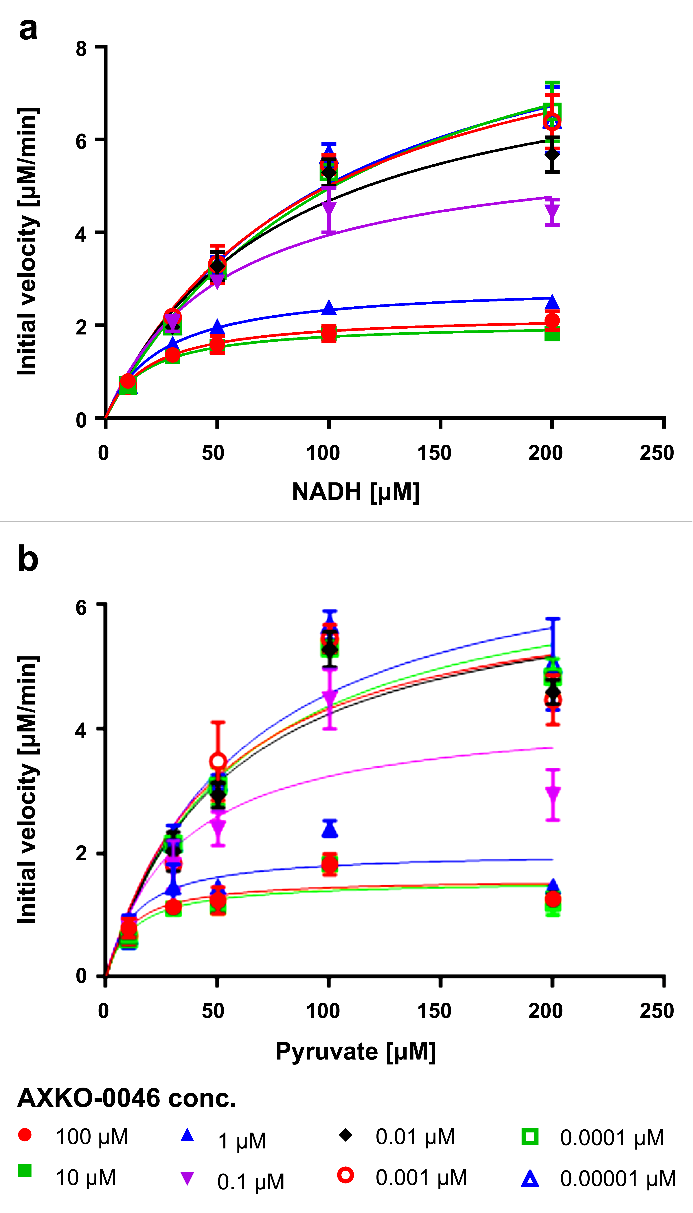
**

**Supplementary Figure 3. Nonlinear fits of the Michaelis–Menten kinetics for LDHB in the presence of AXKO-0046.** Michaelis–Menten kinetics for LDHB were determined from the initial reaction velocities of (**a**) NADH and (**b**) pyruvate at varying substrate concentrations in the presence of the indicated concentration of AXKO-0046.


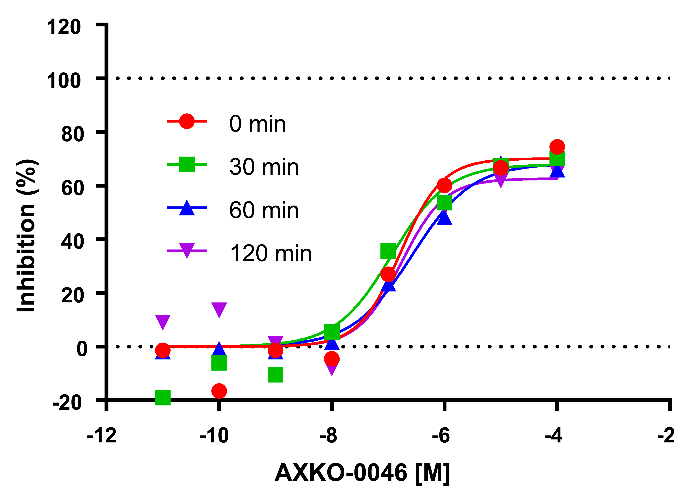


**Supplementary Figure 4. Time-dependent inhibition of LDHB activity by AXKO-0046.** Time-dependent inhibition was measured by pre-incubating AXKO-0046 and LDHB for 0, 30, 60, or 120 min, as indicated, before initiating the LDHB reaction. Data are shown as mean (n = 2).


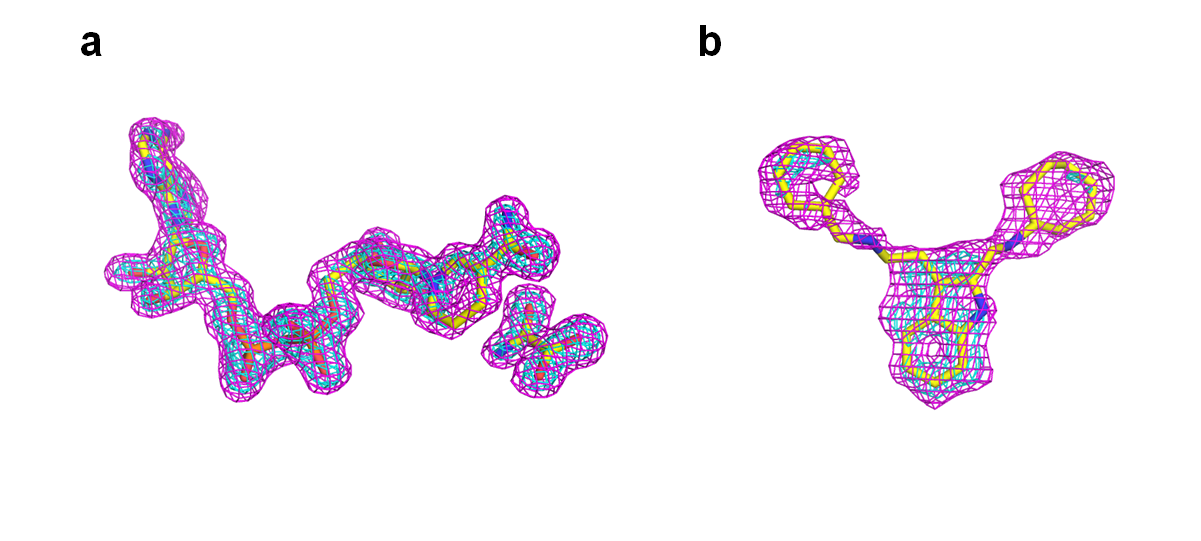


**Supplementary Figure 5. F_obs_-F_calc_ electron density omit maps of the quarterly complex contoured at 3σ**. (**a**) NADH and oxamate and (**b**) AXKO-0046.


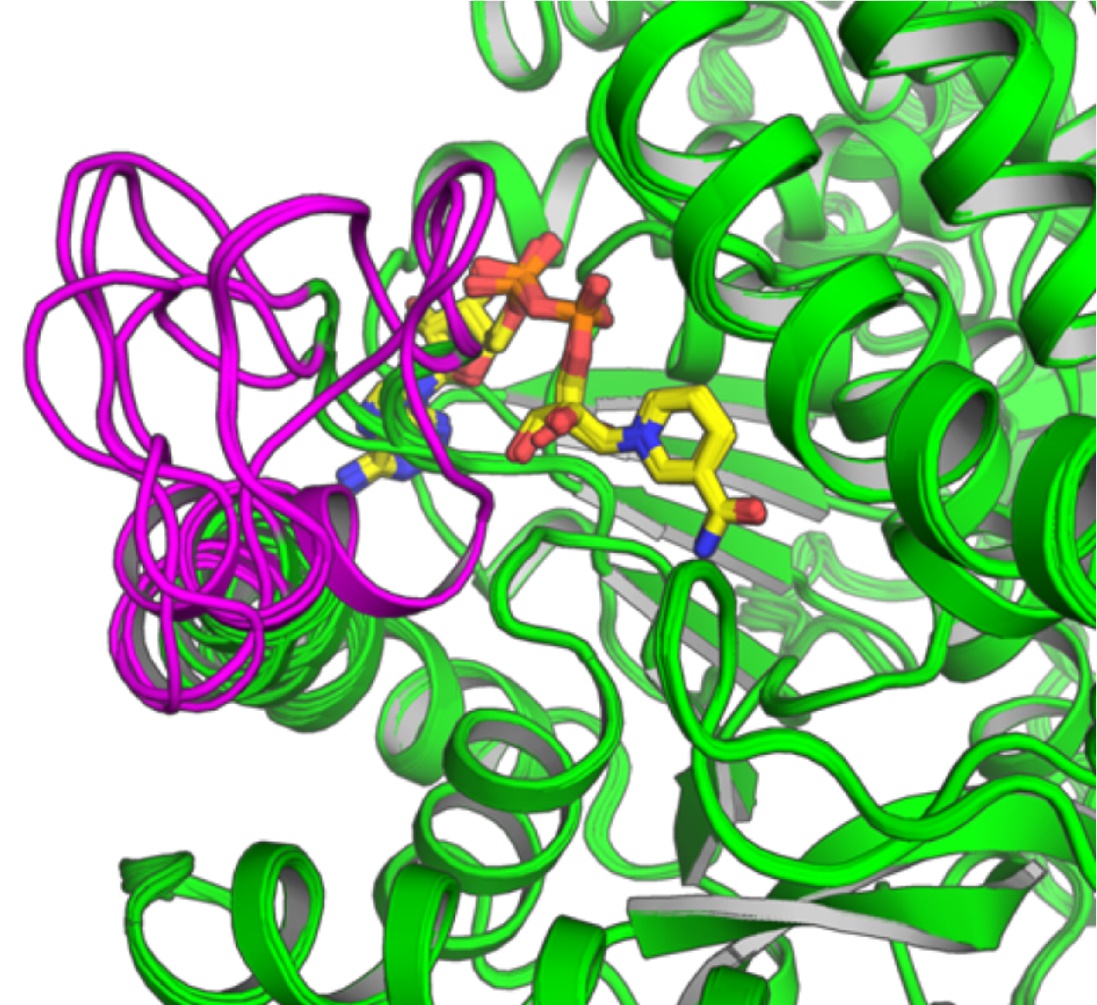


**Supplementary Figure 6. Superposition of eight monomers in the asymmetric unit of the LDHB/NADH complex.** The active-site loop (residues Glu101–Leu110) is in magenta.


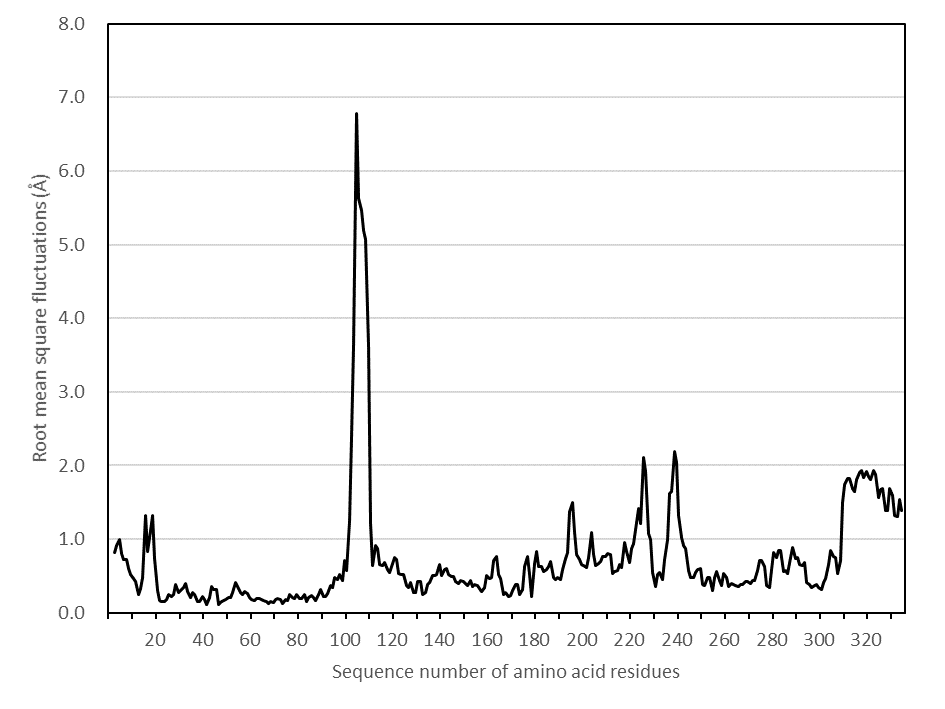


**Supplementary Figure 7. Comparison of the root-mean-square fluctuation values per amino acid residue of LDHB between the two complexes.** RMSF values were calculated from the sets of aligned conformations.


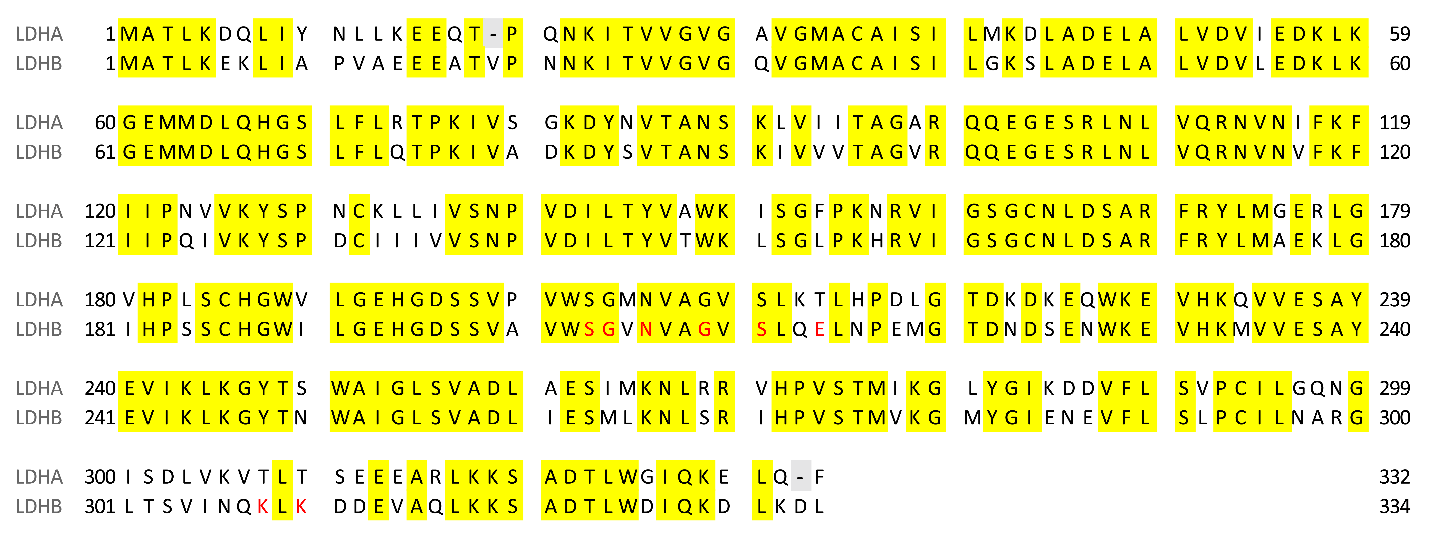


**Supplementary Figure 8. Sequence alignment of human LDHA and human LDHB.** Residues depicted in Figure 5b are in red.


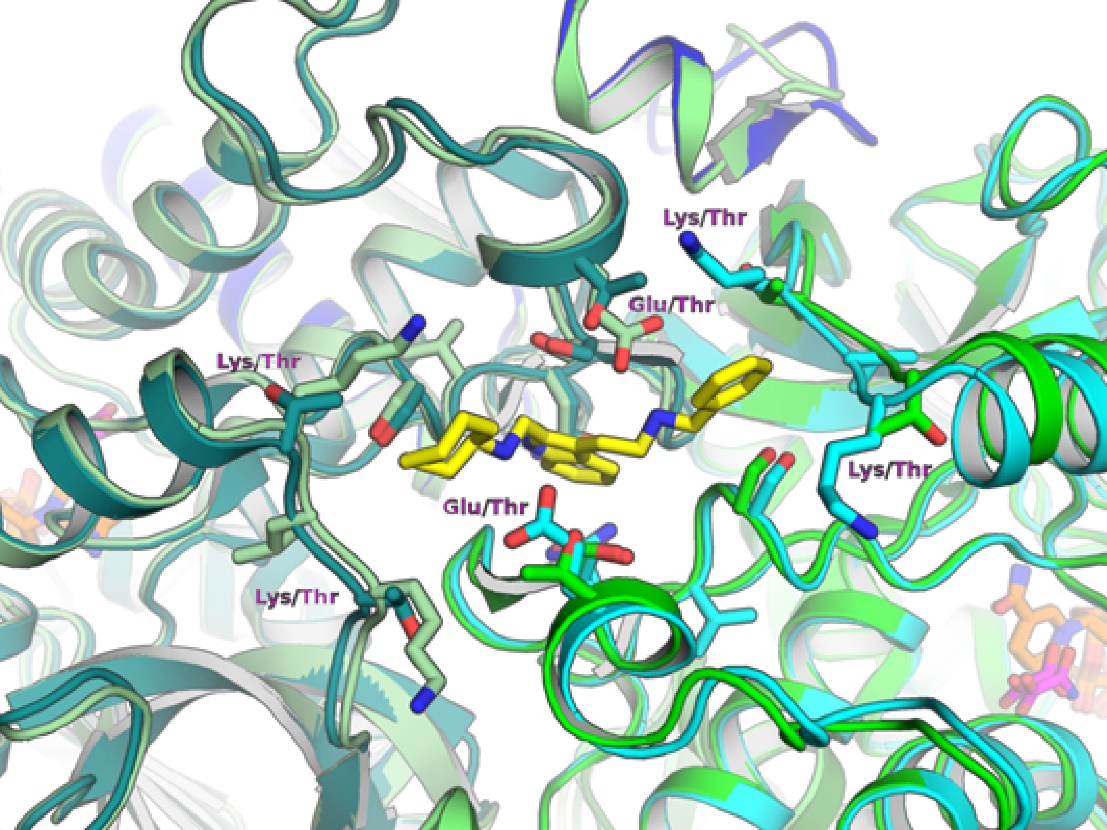


**Supplementary Figure 9. Structural comparison between the allosteric sites of LDHB and LDHA (PDB code 4OKN).** Amino acid differences between enzymes are labelled.

**Supplementary Table 1. Selectivity profile of the LDHB hit compound.**

| ID | Deconvolution assay  (% inhibition at 30 μM) | LDHB  (IC_50_, μM) | LDHA  (IC_50_, μM) | Ratio  (LDHA (IC_50_)  /LDHB (IC_50_)) |
| --- | --- | --- | --- | --- |
| AXKO-0001 | 50 | 23 | 12 | 0.5 |
| AXKO-0002 | 95 | 5.6 | < 3 | < 0.5 |
| AXKO-0003 | 50 | 33 | 71 | 2.2 |
| AXKO-0004 | 45 | 15 | > 300 | 20.0 |
| AXKO-0005 | 38 | 32 | 34 | 1.1 |
| AXKO-0006 | 37 | 30 | 6.4 | 0.21 |
| AXKO-0007 | 40 | 38 | 28 | 0.7 |
| AXKO-0008 | 53 | 16 | 130 | 8.1 |
| AXKO-0009 | 54 | 21 | 39 | 1.9 |
| AXKO-0010 | 44 | < 3 | > 300 | > 100 |
| AXKO-0011 | 59 | 17 | 22 | 1.3 |
| AXKO-0012 | 53 | 8.1 | 6.8 | 0.8 |
| AXKO-0013 | 84 | 5.5 | 30 | 5.5 |
| AXKO-0014 | 67 | 15 | 77 | 5.1 |
| AXKO-0015 | 51 | 30 | 130 | 4.3 |
| AXKO-0016 | 55 | 13 | 19 | 1.5 |
| AXKO-0017 | 60 | 22 | 24 | 1.1 |
| AXKO-0018 | 92 | 6.7 | < 3 | < 0.4 |
| AXKO-0019 | 49 | 27 | 36 | 1.3 |
| AXKO-0020 | 78 | 9.5 | 22 | 2.3 |
| AXKO-0021 | 53 | 20 | 63 | 3.2 |
| AXKO-0022 | 34 | 15 | 39 | 3.2 |

**Supplementary Table 2. Inhibitory activity of LDHB by AXKO-0046 at varying concentrations of NADH and pyruvate.**

|  | NADH (μM) | | | | |
| --- | --- | --- | --- | --- | --- |
|  | 10 | 30 | 50 | 100 | 200 |
| IC_50_ (nM) | N.D. | 702.1 | 329.5 | 223.0 | 94.2 |
|  | Pyruvate (μM) | | | | |
|  | 10 | 30 | 50 | 100 | 200 |
| IC_50_ (nM) | N.D. | 197.1 | 114.2 | 223.0 | 96.3 |

N.D.: not determined.

**Supplementary Table 3. Data collection and refinement statistics.**

|  | LDHB/NADH/oxamate/AXKO-0046 | LDHB/NADH |
| --- | --- | --- |
| **Data collection** |  |  |
| Space group | *P*2_1_ | *C*2 |
| Unit cell dimensions a, b, c (Å), α, β, γ (°) | 59.4, 137.6, 84.9,  90, 109.3, 90 | 232.5, 84.2, 156.2,  90, 120.8, 90 |
| Resolution (Å) | 50-1.55 (1.58-1.55) | 50-1.80 (1.83-1.80) |
| Redundancy | 4.1 (3.5) | 3.4 (3.4) |
| Completeness (%) | 99.2 (93.8) | 99.6 (100.0) |
| I/σ | 20.3 (4.0) | 19.8 (2.1) |
| R_sym_ | 0.076 (0.251) | 0.065 (0.511) |
| CC_1/2_ | 0.989 (0.923) | 0.994 (0.736) |
| **Refinement** |  |  |
| Resolution (Å) | 50-1.55 (1.58-1.55) | 50-1.80 (1.85-1.80) |
| No. reflections | 174218 (12517) | 225765 (17284) |
| R_work_/R_free_ | 0.170 (0.204) / 0.192 (0.233) | 0.170 (0.238) / 0.203 (0.269) |
| No. atoms  Protein / Ligand / Water | 10253 / 284 / 765 | 20488 / 484 / 977 |
| B-factors (Å^2^)  Protein / Ligand / Ion / Water | 25.2 / 28.7 / 32.5 | 31.5 / 31.0 / 32.4 |
| Rms deviation from ideal geometry  Bond lengths (Å) / angles (°) | 0.011 / 1.549 | 0.010 / 1.548 |
| Ramachandran plot  Preferred / Allowed / Outliers (%) | 96.1 / 3.2 / 0.7 | 94.7 / 4.6 / 0.7 |
| **PDB code** | 7DBJ | 7DBK |

Values in parentheses indicate the highest-resolution shell.
